# Stage-Related Neurotoxicity of BPA in the Development of Zebrafish Embryos

**DOI:** 10.3390/toxics11020177

**Published:** 2023-02-14

**Authors:** Jianjun Liu, Wenyu Kong, Yuchen Liu, Qiyao Ma, Qi Shao, Liwen Zeng, Yu Chao, Xiaoyao Song, Jie Zhang

**Affiliations:** Department of Toxicology, School of Public Health, Medical College of Soochow University, Suzhou 215031, China

**Keywords:** bisphenol A, zebrafish, sensitive period, guanine deaminase, locomotor behavior

## Abstract

Bisphenol A (BPA) is one of the most widely produced chemicals in the world used in the production of epoxy resins and polycarbonate plastics. BPA is easily migrated from the outer packaging to the contents. Due to the lipophilic property, BPA is easily accumulated in organisms. Perinatal low-dose BPA exposure alters brain neural development in later generations. In this study, after BPA treatment, the spontaneous movement of zebrafish larvae from the cleavage period to the segmentation period (1–24 hpf) was significantly decreased, with speed decreasing by 18.97% and distance decreasing between 18.4 and 29.7% compared to controls. Transcriptomics analysis showed that 131 genes were significantly differentially expressed in the exposed group during the 1–24 hpf period, among which 39 genes were significantly upregulated and 92 genes were significantly downregulated. The GO enrichment analysis, gene function analysis and real-time quantitative PCR of differentially expressed genes showed that the mRNA level of guanine deaminase (cypin) decreased significantly in the 1–24 hpf period. Moreover, during the 1–24 hpf period, BPA exposure reduced guanine deaminase activity. Therefore, we confirmed that cypin is a key sensitive gene for BPA during this period. Finally, the cypin mRNA microinjection verified that the cypin level of zebrafish larvae was restored, leading to the restoration of the locomotor activity. Taken together, the current results show that the sensitive period of BPA to zebrafish embryos is from the cleavage period to the segmentation period (1–24 hpf), and cypin is a potential target for BPA-induced neurodevelopmental toxicity. This study provides a potential sensitive period and a potential target for the deep understanding of neurodevelopmental toxicity mechanisms caused by BPA.

## 1. Introduction

Bisphenol A (BPA) is one of the most widely produced chemicals in the world, as an analogue of bisphenol (BP). BPA is mainly used in industry to synthesize materials such as polycarbonate and epoxy resin. It has been used in the manufacture of plastic cups, baby bottles, food and beverage cans since the 1960s [1,2,3,4]. Studies have shown that BPA is easily migrated from containers or packaging materials to food and beverages. Due to the lipophilic property (LogK_ow_ = 3.3), BPA is easily accumulated in organisms. BPA has been detected in wild animals, especially fish (0.2–13,000 ng/g) [5], and can be detected in human blood and urine. A study reported that BPA was found in 46% of all blood samples analyzed, with a geometric mean (GM) concentration of 0.19 ng/mL. BPA was found in 84% of urine samples from adults, with a GM concentration of 1.01 ng/mL [0.48 μg/g creatinine (Cr)] [6,7,8]. According to reports, a significant positive correlation was found between serum and urine BPA levels in pregnant women and neonates [9,10]. Exposure to BPA during pregnancy induced anxiety, reduced exploratory behavior and increased depressive behavior in adult mice [11]. Similarly, perinatal low-dose BPA exposure has altered brain neural development in offspring rats [12,13].Therefore, exposure to BPA in the early stages of life is of great concern, in which the embryo and infancy stage play a very important role in the natural development of the nervous system.

Zebrafish (Danio rerio) is a model organism widely used in toxicological evaluation [14,15,16]. Recent reports have shown that brain structures are homologous between zebrafish and humans such as the amygdala, hippocampus and hypothalamus [17]. Furthermore, zebrafish show a wide range of complex behavior in cognition, aggression, anxiety and social interaction [18]. Thus, zebrafish is a useful tool for elucidating the function of novel genes for neurogenesis [19] and have been used to validate the function of human candidate genes involved in autism, etc. [19,20,21]. After fertilization, zebrafish embryos go through seven stages to complete embryonic development [22]: the zygotic stage (0–0.75 hpf), cleavage period (0.75–2.20 hpf), blastula period (2.20–5.25 hpf), gastrula period (5.25–10 hpf), segmentation period (10–24 hpf), pharyngeal period (24–48 hpf) and hatching period (48–72 hpf). The nervous system starts to develop and form the neural plate at 6 hpf, and the neural tube and different brain regions form beginning in the 10–16 hpf period. By 24 hpf, brain morphogenesis is advanced, and the brain is divided into the forebrain, including the diencephalon and telencephalon, midbrain, hindbrain and spinal cord, while the earliest clusters of neurons are interconnected by axons [23]. There is a correspondence between the embryonic and the behavior development of zebrafish. The spontaneous contractions of trunk muscles of zebrafish can be observed at 17 hpf, tail movement appears at 21 hpf, the locomotor ability of zebrafish shows the avoidance reflex phenomenon to external stimuli at 48 hpf and the rate of swimming in response to touch becomes maximal at 36 hpf close to that of adult zebrafish [24,25]. Therefore, damage to the nervous system often results in changes in behavior [26]. Normally developed zebrafish embryos have the ability to move freely at 96–120 hpf, and their locomotor ability can be used as a sensitive endpoint for exogenous chemicals to damage the nervous development of the body [27].

In this study, we integrated transcriptomics and neurodevelopmental toxicity analysis to comprehensively study the potential biological mechanisms of BPA exposure in zebrafish during the embryonic development period. We investigated the BPA-induced neurotoxicity in different stages of the development of zebrafish embryos to reveal the possible sensitive period of the development upon exposure to BPA and to explore the potential biomarker of BPA neurodevelopmental toxicity.

## 2. Materials and Methods

### 2.1. Chemicals and Reagents Preparation

BPA powder (99%) was purchased from Sigma-Aldrich (St Louis, MO, USA). A stock solution of BPA was dissolved and diluted in dimethyl sulfoxide (DMSO) (Sigma-Aldrich, St Louis, MO, USA) to achieve BPA treatment concentrations. DEPC water (Beyotime, Nanjing, China) was used when cleaning zebrafish embryos and diluting cDNA.

### 2.2. BPA Exposure of Zebrafish Embryos

**Figure 1:**
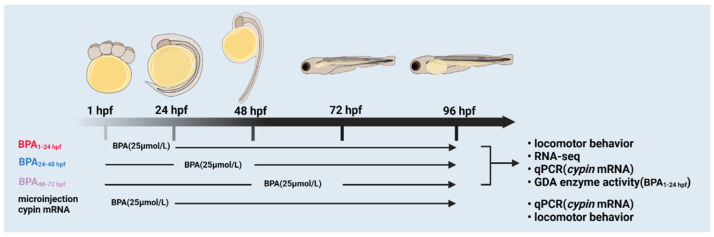
A workflow of the present study design.

**Figure 2:**
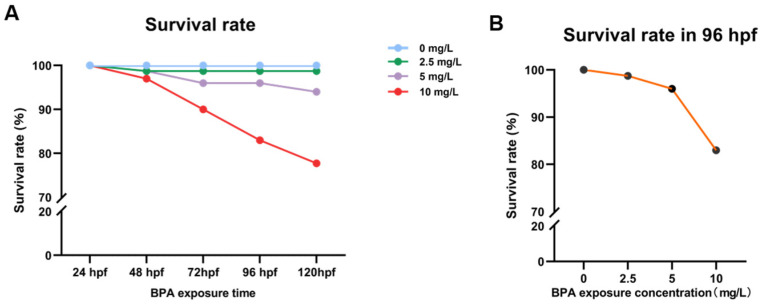
Survival rate of zebrafish exposed to BPA. (**A**) Survival rate of zebrafish exposed to different concentrations of BPA. (**B**) Survival rate of zebrafish exposed to different concentrations of BPA at 96hpf. The modified Kohl method was used to calculate the LC_50, 72 hpf_ = 12.639 mg/L, 95% confidence interval of 11.857–13.473; LC_50, 96 hpf_ = 10.193 mg/L, 95% confidence interval of 9.040–11.494; LC_50, 120 hpf_ = 9.045 mg/L, 95% confidence interval of 7.608–10.753. Data are the mean ± SD (n = 90 in each concentration).

The workflow of the present research is in Figure 1. Zebrafish (Danio rerio) were obtained from the Zebrafish Experiment Center of Soochow University (Suzhou, China). They were raised in standard laboratory conditions at 28.5 °C with a 14:10 light/dark (LD) cycle. Zebrafish were fed three times per day. Male and female adult fish (male/female = 1/1 or 1/2) were separated by isolation boards in spawning tanks in the evening. The isolation boards were removed when the light turned on automatically the next morning; then, the embryos were collected within 2 h and rinsed with fish culture water for subsequent experiments. Healthy embryos were selected by stereomicroscopy. In total, 30 embryos per group were placed in an empty well of a 6-well plate, and then 3 mL of systemic culture water was added to each well. According to the characteristics of zebrafish embryo development, each stage of early embryo development was completed in 96 hpf, so the time point of 96 hpf was selected in the follow-up experiment. According to 96 hpf median lethal concentration, we determined that the LC_50_ was 11.4 mg/L. As design, we chose half of the LC_50_ (5.7 mg/L) as the exposure concentration. Zebrafish embryos were treated with 5.7 mg/L BPA (Figure 2) from the cleavage period to the segmentation period (1–24 hpf), pharyngula period (24–48 hpf) and hatching period (48–72 hpf); washed with fish culture water 3 times and cultured until 96 hpf (changing water every day). The DMSO solvent control (SC) group and blank control (BC) group were set up (the final concentration of DMSO was 0.1%). Each dose group had three replicates. The embryos were chorionated. The dead embryos were picked out during the culture process. All animal protocols were performed in accordance with the Guidelines on the Care and Use of Animals and with the approval of the Soochow University Animal Welfare Committee.

### 2.3. Locomotor Behavioral Analysis

To investigate the effect of early-life exposure to BPA on the nervous system, we studied the locomotor behavioral change underlying two scenarios such as spontaneous motion and light-dark cycle stimulation. Three experimental replicated with 30 embryos were exposed. Excluding embryos that died or that had overt toxicity, we selected 8 zebrafish for each treatment group. Statistics were calculated for at least 8 zebrafish per treatment group; then, the differences in 3 replicate experiments were calculated. At 96 hpf, the normally developed zebrafish larvae were placed in a 96-well plate, one for each well, and 250 μL of systematic fish culture water was added to make sure the zebrafish larvae could swim freely. The 96-well plate was placed in a Zebralab high-throughput behavior analyzer (View PointLife Sciences, Lyon, France) to detect the changes in locomotor behavior.

For spontaneous motion detection, after the light adapting to 30 min in the system, the spontaneous movement of zebrafish larvae within 10 min was collected, and the swimming speed of zebrafish larvae was calculated automatically.

For the light-dark cycle stimulus assay, after the light adapting to 30 min in the system, the light–dark cycle was set to 90 s (90 s light/90 s dark alternating) and repeated 3 times to detect the response of zebrafish to the dark-light transition stimulus.

### 2.4. Transcriptomics Analysis

In order to explore the toxic mechanism of BPA in the sensitive stage of neurotoxicity, we used RNA-seq to analyze the transcriptome of zebrafish larvae in SC group and exposed group. The total genomic RNA of zebrafish exposed to BPA from the cleavage period to the segmentation period (1–24 hpf) was extracted. The cracking reagent was blown, mixed and centrifuged (13,000 rpm, 4 °C) to obtain short mRNA. The short mRNA was used as the template to synthesize cDNA1 chain, and then the cDNA2 chain was further synthesized and purified. Then, the poly A tail was added to the end of the cDNA2 chain, and the joint sequence was connected. The DNA fragment was enriched after purification and analyzed by a biological analyzer. The RIN (RNA Integrity Number) value was 8 after analyzing on the bioanalyzer. The library was constructed, and the quality inspection was carried out. The screening of differential genes was performed to calculate the read number of genes in each sample according to htseq-count software. After obtaining the raw reads in FastQ format, the 3′ end adapter sequence was pruned, and the low-quality value (<20) region in the sequencing files was removed. Gene expression level was expressed by fragments per kilobase of transcript per million mapped reads (FPKM). The raw read counts were used to analyze differentially expressed genes (DEGs) using the “DESeq” package. Then, the data were standardized, the differential genes were selected according to the *p* value (*p* < 0.05) and fold change values larger than 1.5 of the differences, unsupervised hierarchical clustering of differential genes was performed. GO enrichment analysis of the differentially expressed genes was carried out to show the function of the differentially expressed genes.

### 2.5. Real-Time Quantitative PCR Analysis

Total RNA was isolated from larvae using TRIzol reagent (Beyotime). The yields of RNA samples were measured with a Nanodrop spectrophotometer (ThermoFisher Scientific, Waltham, MA, USA). All samples had an OD A_260_/A_280_ ratio (range 1.8–2.0). A total of 1000 ng of RNA was used for the cDNA synthesis reaction using a RevertAid First Strand cDNA Synthesis Kit (ThermoFisher Scientific). Quantitative real-time PCR analysis was carried out with QuantStudio 6 (ThermoFisher Scientific) using the maxima SYBR Green qPCR Master Mix (Roche), and subjected to the following two-step RT-PCR method: 120 s at 95 °C, followed by 45 cycles of 95 °C for 20 s and 60 °C for 40 s. The transcription of β-actin was used as a housekeeping gene. Gene expression levels were measured in triplicate for each treatment. The sequences of the primers used in this study are presented in Table S1. The fold change of genes tested was calculated using the 2^−△△CT^ method.

### 2.6. Microinjection

Based on changes in mRNA activity in the results of the above transcriptomics analysis and real-time quantitative PCR analysis, we focused on Cypin. In order to verify the role of cypin in BPA exposure, we used rescue experiments. At the position of about 300 bp after polyA, the primer sequence of the target gene cypin was designed with the vector (pCDNA3.1-HA), restriction site (Notl, Nhel) and target gene (cypin). The total RNA of the brain tissue of zebrafish was used as template and reverse transcribed into cDNA as template for PCR amplification. Zebrafish cypin fragments were amplified by designed primer sequences and analyzed by agarose gel electrophoresis. After gel recovery and homologous recombination, the recombinant plasmid was digested with Notl and Nhel, and the sequencing results showed that cypin was cut by agarose gel electrophoresis. After sequencing, it was found that the sequenced recombinant plasmid was the target gene cypin plasmid, indicating that the plasmid was constructed successfully. After cypin sequencing, the promoter was amplified with T7 promoter and pCDNA3.1-HA as upstream and downstream primers. The PCR product was purified, and the mRNA of cypin was synthesized by T7 transcriptase. Agarose gel electrophoresis analysis showed that the transcription was successful. All zebrafish eggs were divided into six groups: blank control group (BC group); microinjection of DEPC water group (DEPC group); microinjection of cypin mRNA group (cypin group); BPA treatment group; BPA treatment after microinjection of DPEC water group (BPA + DEPC group); and BPA treatment after microinjection of cypin mRNA group (BPA + cypin group). The BPA treatment group, BPA + DEPC group and BPA + cypin group were exposed to BPA for 24 hpf from the cleavage period to the segmentation period (1–24 hpf). The purified mRNA was diluted to a 0.3 M injection sample with Rnase-free water. The injection pressure was adjusted so that the volume of each injection was 1–1.5 nL, and cypin was injected in the single-cell stage of zebrafish embryos. After washing with fish culture water 3 times, they were cultured to 96 hpf (changing water every day). Triplicates were set up at all concentrations, and dead fish embryos were selected in time during culture. Zebrafish behavior and qPCR analysis experiments were carried out after 96 hpf.

### 2.7. Guanine Deaminase Activity Assay

The guanine deaminase activity of zebrafish exposed to BPA (5.7 mg/L) from the cleavage period to the segmentation period (1–24 hpf) and of the blank control group were detected using a guanine deaminase kit (Kanglang Biotechnology, Shanghai, China). The absorbance (OD) was determined by a microplate reader (BIO-TEX, Southlake, TX, USA) at 450 nm, and the content of guanine deaminase (GDA) in zebrafish samples was calculated by a standard curve. Taking the concentration of the standards as the abscissa and the OD value as the ordinate, and the corresponding concentration was determined from the standard curve according to the OD value of the sample and then multiplied by the dilution multiple, that is, the actual concentration of the sample.

### 2.8. Statistical Analysis

A formal acute toxicity test was designed using the modified Kohl method (Kaber) [28]. The movement speed and distance of zebrafish were counted by EthoVisionXT10.0 analysis software (Noldus Information Technologies, Wageningen, The Netherlands), and the data were collected once a second. The experimental data were statistically analyzed by SPSS 17.0 software (IBM, Armonk, NY, USA), and the statistical results were expressed as the mean ± SD. In the rescue experiment, one-way analysis of variance (one-way ANOVA) corrected by Bonferroni was used for multiple comparisons. Guanine deaminase activity was tested by a t-test. In addition, *p*-values less than 0.05 was considered as statistical difference.

## 3. Results

### 3.1. Survival Rate of Zebrafish Exposed to BPA

Based on previous studies, embryos were exposed to BPA concentrations of 0, 2.5, 5 and 10 mg/L from 0 to 96 hpf, which is when embryos have crossed the hatching stage and completed early embryo development. In total, 30 zebrafish embryos were in each dose group, with 3 replicates. After that, the embryo survival rate was recorded from 24 hpf up to 120 hpf after BPA exposure (Figure 2A). According to the characteristics of zebrafish embryo development, 96 hpf zebrafish embryos have crossed the hatching stage and completed early embryo development; therefore, during the follow-up experiment, we determined 96 hpf as the end of the time of exposure. According to the LC_50, 96 hpf_, the subsequent experimental concentration was determined to be about half of the median lethal concentration, that is, 5.7 mg/L.

### 3.2. Effects of BPA on Zebrafish Locomotor Behavior

**Figure 3:**
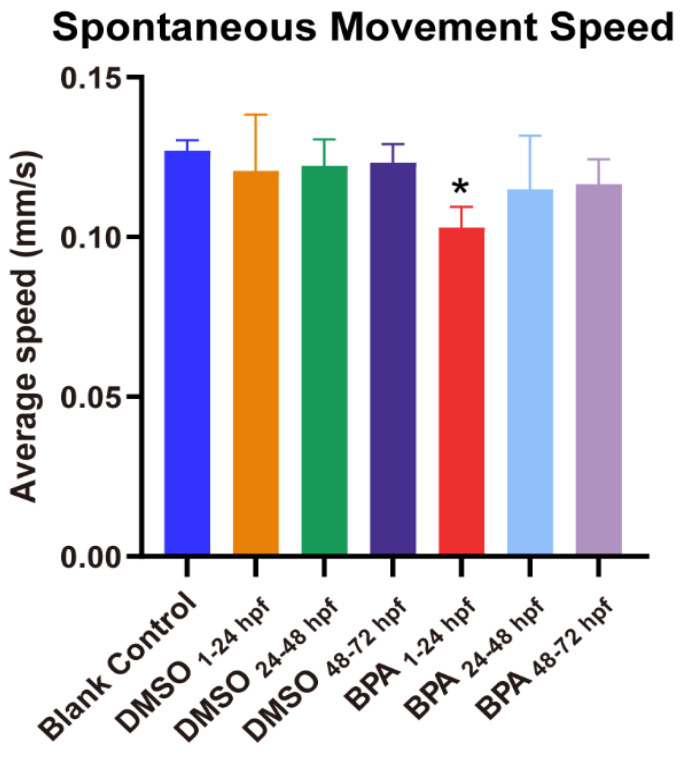
Effects of exposure to BPA on spontaneous movement speed of zebrafish embryos at different developmental periods. Data are the mean ± SD (n = 24 in each concentration). *, significant from blank control, *p* < 0.05.

**Figure 4:**
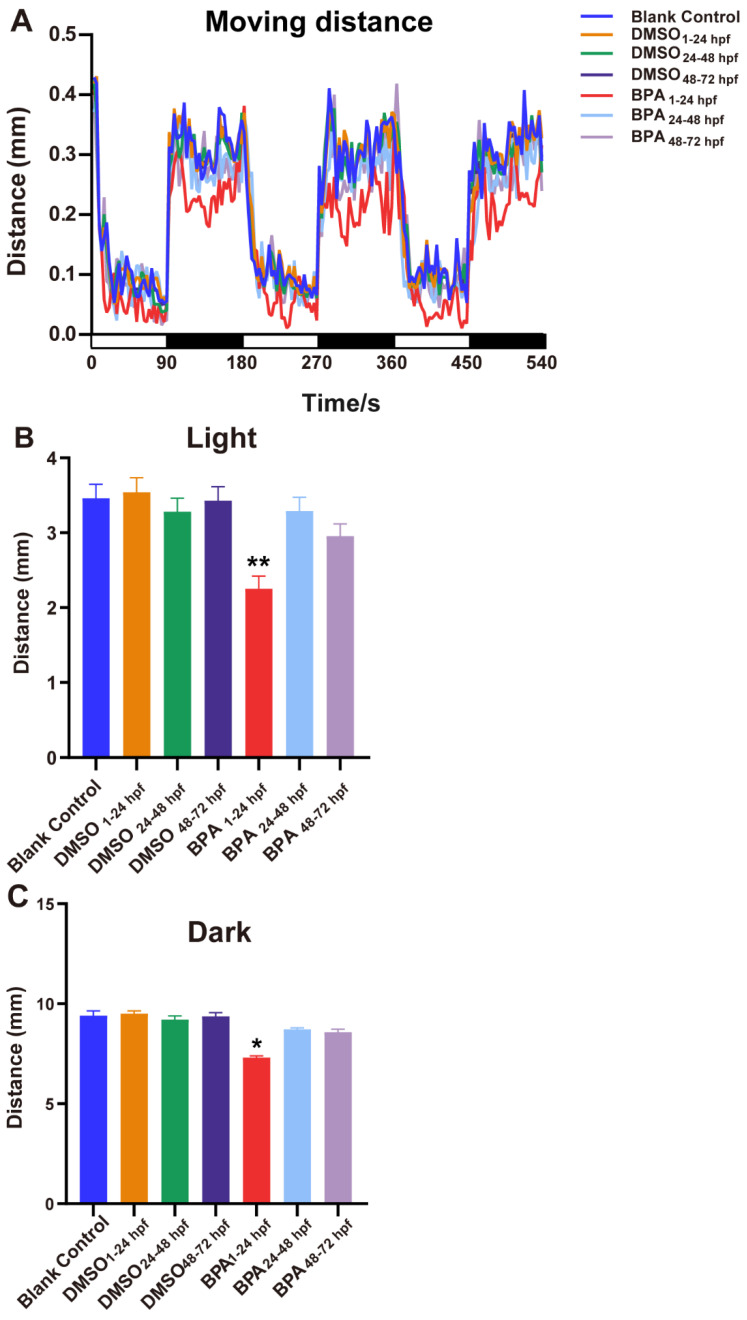
Effects of exposure to BPA on locomotor behavior under the light-dark cycle stimulation of zebrafish embryos at different developmental periods. (**A**) The trend of distance. (**B**,**C**) The analysis results of the trend of the distance. Abscissa white for light, black for darkness. Data are the mean ± SD (n = 24 in each concentration). *, significant from blank control, *p* < 0.05, **, significant from blank control, *p* < 0.01.

As shown in Figure 3, the spontaneous movement speed of zebrafish in the blank control group was approximately 0.13 mm/s, while that in the exposed group from the cleavage period to the segmentation period (BPA_1–24hpf_ group) decreased significantly (*p* < 0.05), at approximately 18.97% lower than control. Compared with the BC group, there were no statistical differences of spontaneous movement speed in other BPA-exposed-period groups (*p* > 0.05). Generally, larvae increase their movement when subjected to dark conditions. This increase in movement was consistent across multiple dark-light cycles (Figure 4A). However, further analysis found that from the cleavage period to the segmentation period (1–24 hpf), the moving distance in the light-dark cycle of the BPA exposure group of zebrafish larvae were significantly decreased, dropping 29.7% in the light period (*p* < 0.05, Figure 4B) and 18.4% in the dark period (*p* < 0.05, Figure 4C). There were no statistical differences in the light-dark cycle in the other groups compared with the BC group. Therefore, we confirmed that the sensitive period of BPA on the locomotor behavior of zebrafish larvae was from the cleavage to the segmentation period (1–24 hpf) in this study.

### 3.3. Transcriptome Analysis of Zebrafish Larvae at Different Developmental Periods after BPA Exposure

**Figure 5:**
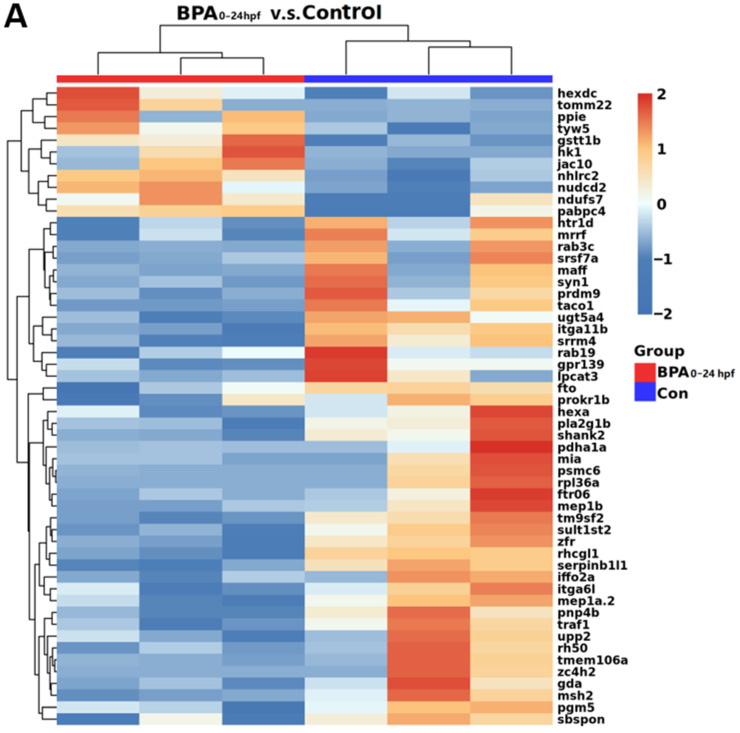

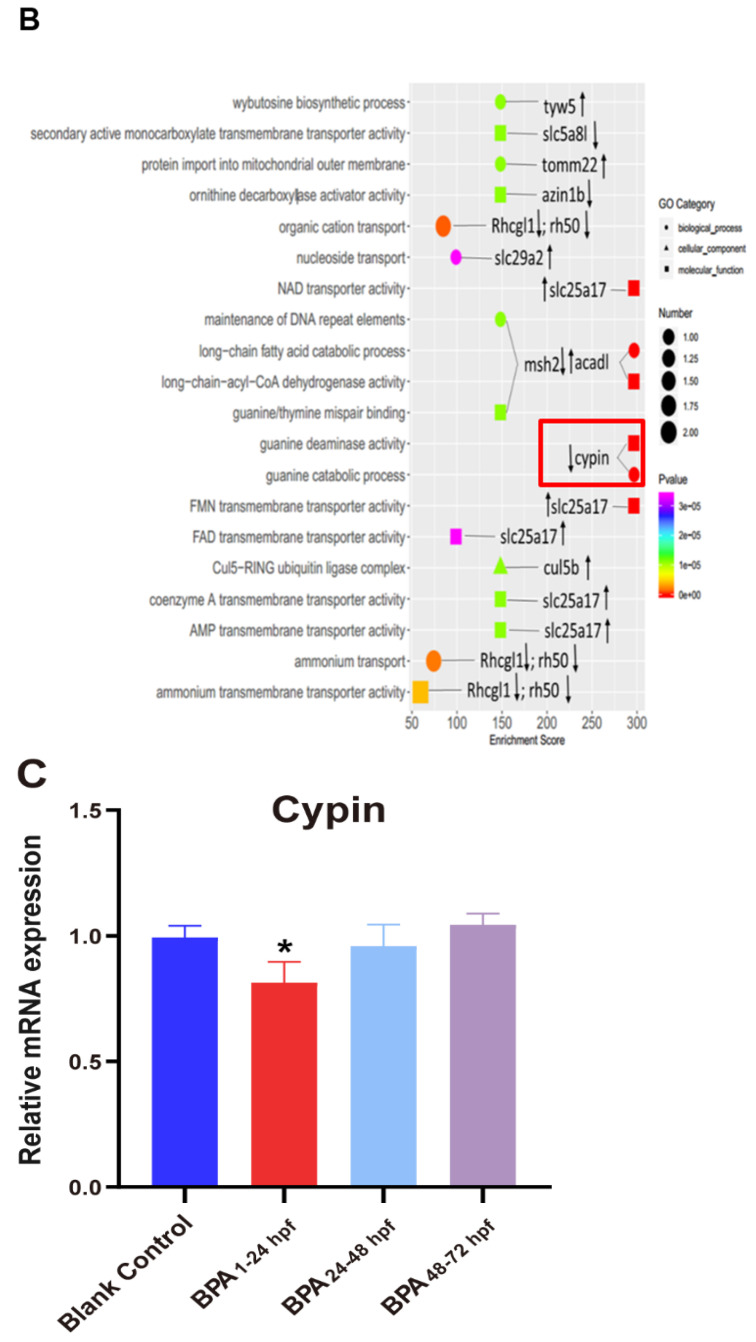
Transcriptome analysis of zebrafish from the cleavage period to the segmentation period (1–24 hpf), pharyngula period (24–48 hpf) and hatching period (48–72 hpf) induced by BPA. (**A**) Cluster analysis heatmap. Red indicates increased gene expression, and blue indicates decreased gene expression. (**B**) GO function enrichment analysis bubble diagram for differentially expressed genes. (**C**) The expression of cypin. Data are the mean ± SD (30 embryos per sample, and repeat three times), *, significant from blank control, *p* < 0.05.

Figure 5A shows the hierarchical cluster heatmaps of differential genes expression based on log10 of FPKM values through cluster analysis. As shown in Figure 5A, compared with the BC group, there were statistical differences in 131 differential genes in the BPA-exposed group during 1–24 hpf, of which 39 were significantly upregulated and 92 were significantly downregulated (Figure 5A). As shown in Figure 5B, we screened the differential genes of the cleavage period to the segmentation period (1–24 hpf) in the exposed group using the gene ontology (GO) analysis. Then, we listed the enrichment scores of the top 20 terms that corresponded to the differential genes. Among them, there were six entries with the highest enrichment scores corresponding to three GO terms: long-chain fatty acid decomposition in the process of biology (acadl); cypin in the guanine catabolic processes; molecular functions, including NAD transporter activity and FMN transporter activity (slc25a17), long-chain acyl coenzyme A dehydrogenase activity (acadl) and guanine catabolism (cypin). Because of the enrichment score of cypin and its enrichment in both terms, and because it was closely related to neural development, we included it as a biomarker for further investigation. As shown in Figure 5C, the results of real-time qPCR showed that the expression of cypin was significantly decreased after exposure to BPA from the cleavage period to the segmentation period (1–24 hpf) compared with the BC group (*p* < 0.05). Our transcriptome sequencing results also showed that cypin mRNA levels of 1–24 hpf zebrafish larvae were significantly downregulated in the exposed group. These results suggest that cypin might play an important role in the neural development from cleavage period to the segmentation period (1–24 hpf) of zebrafish.

### 3.4. Changes in Cypin during Zebrafish Embryonic Development

**Figure 6:**
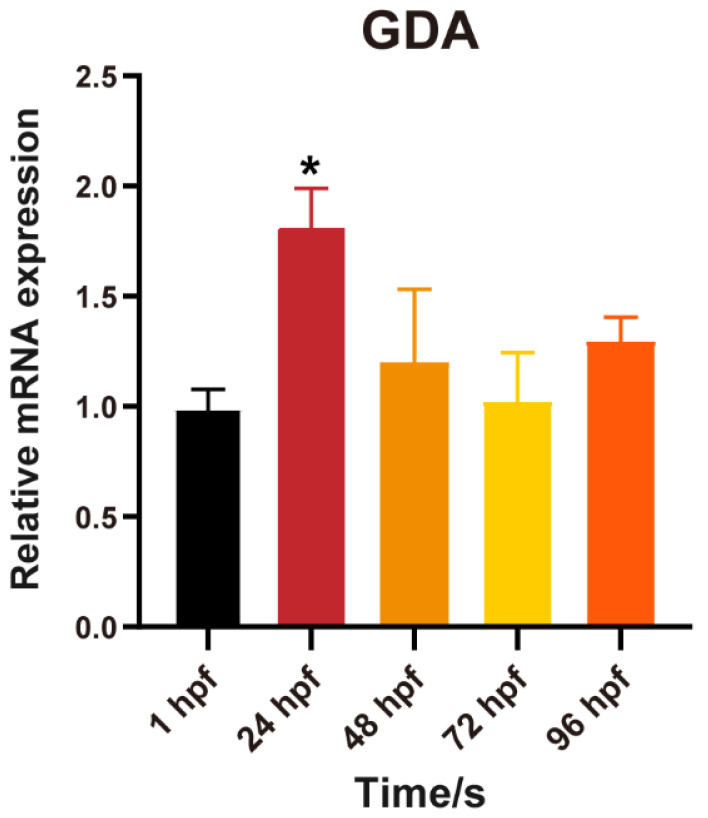
The expression of cypin at different developmental periods. Data are the mean ± SD, *, significant from 1 hpf, *p* < 0.05.

Figure 6 shows the expression of cypin in zebrafish embryos (wild-type unexposed embryos) during development (1 hpf, 24 hpf, 48 hpf, 72 hpf, 96 hpf). The results showed that, compared with 1 hpf zebrafish embryos, the expression of cypin was significantly increased at 24 hpf (*p* < 0.05) and then returned to the 1 hpf level at other points in development time. This suggests that cypin played an important role in early neural development.

### 3.5. Effect of BPA on the Activity of Guanine Deaminase in Zebrafish Embryos during 1–24 hpf

**Figure 7:**
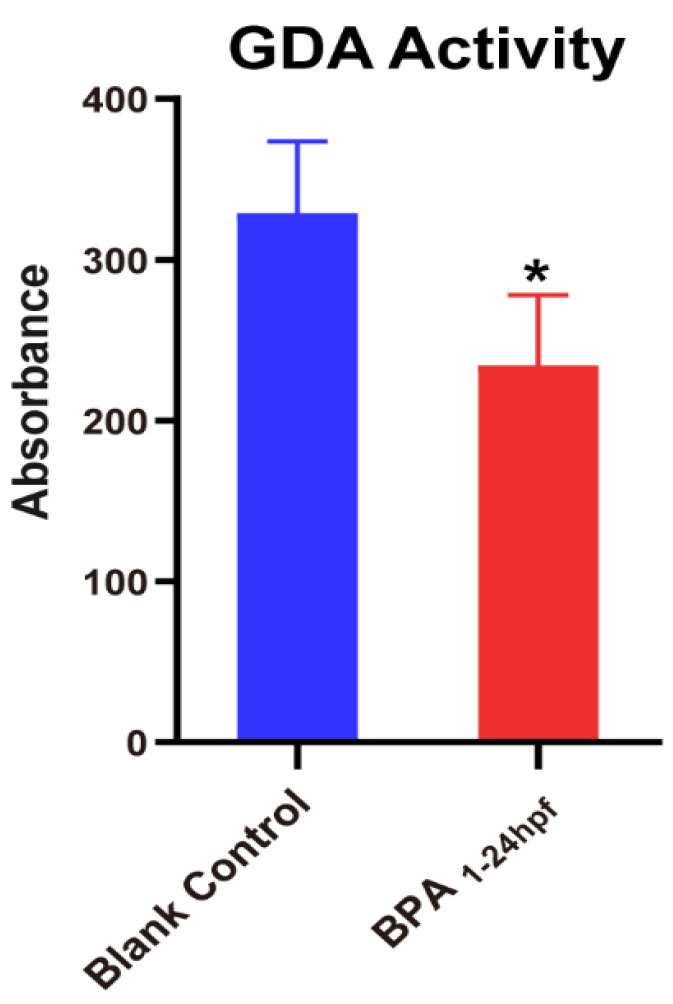
Effects of exposure to BPA on GDA enzyme activity in zebrafish embryos from the cleavage period to the segmentation period (1–24 hpf). Data are the mean ± SD, *, significant from blank control, *p* < 0.05.

Cypin, also known as guanine deaminase (GDA), the primary guanine deaminase in the brain, plays key roles in shaping neuronal circuits and regulating neuronal survival. Based on the above results, we found that the mRNA of cypin only decreased significantly in the stage from the cleavage period to the segmentation period (1–24 hpf). However, in the aforementioned BPA-unexposed zebrafish (Figure 6), the mRNA levels of cypin were significantly elevated at 1–24 hpf compared to those in other stages. The magnitude of cypin change was significant. Therefore, we focused on the changes of GDA activity in BPA exposure stage from the cleavage period to the segmentation period (1–24 hpf). As shown in Figure 7, since zebrafish exposed to BPA at 1-24 hpf had reduced cypin expression, we then tested whether the fish also had reduced GDA and found that the fish exposed to BPA had significantly lower GDA.

### 3.6. Changes in Cypin Expression after Microinjection of Cypin mRNA

**Figure 8:**
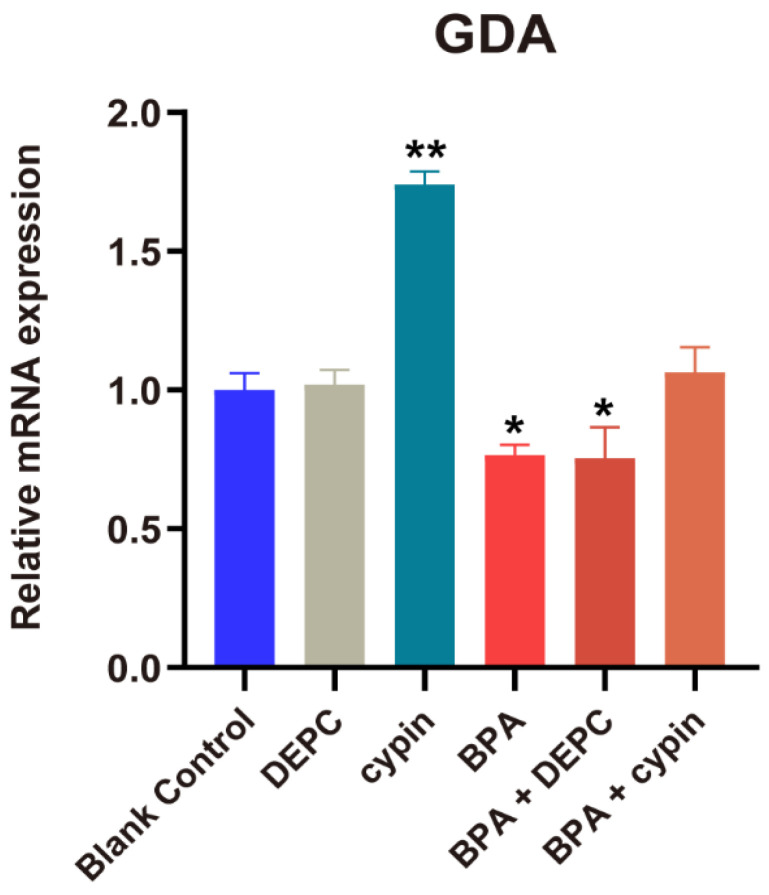
Expression of cypin gene after microinjection of cypin mRNA in zebrafish embryos. Data are the mean ± SD, *, significant from blank control, *p* < 0.05. **, significant from blank control, *p* < 0.01. DEPC group: microinjection of DEPC water; cypin group; microinjection of cypin mRNA; BPA treatment group: BPA treatment; BPA + DEPC group: BPA treatment after microinjection of DPEC; BPA + cypin group: BPA treatment after microinjection of cypin mRNA.

Through the above experiments, we found that BPA exposure reduced the mRNA level and activity of cypin. Therefore, we wanted to confirm whether cypin was the key sensitive gene of BPA during this period by the microinjection of cypin mRNA. After the microinjection of cypin mRNA, we determined the post-injection results of mRNA using qPCR. According to Figure 8, the expression of cypin in the cypin group was significantly higher than that in the BC group. The expression of cypin in BPA treatment group and BPA+DEPC group decreased significantly compared with the BC (blank control) group, but there was no significant difference between the BC group and BPA+ cypin group, suggesting that BPA can induce the downregulation of cypin gene expression and the microinjection of cypin mRNA can reverse the downregulation of cypin gene expression induced by BPA.

### 3.7. The Locomotor Behavior Change of Zebrafish Larvae after Microinjection of Cypin mRNA

**Figure 9:**
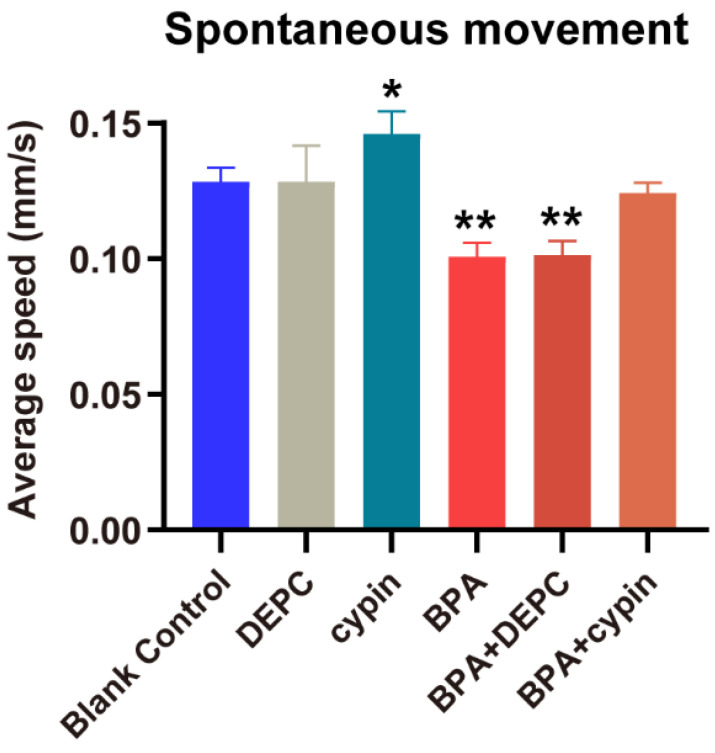
Effect of BPA on the spontaneous movement of zebrafish embryos after microinjection of cypin mRNA. Data are the mean ± SD (n = 24 in each concentration). *, significant from blank control, *p* < 0.05. **, significant from blank control, *p* < 0.01.

After the microinjection of cypin mRNA, the spontaneous movement of zebrafish larvae was observed (Figure 9). Compared with the BC group, there was no statistical difference of spontaneous movement speed in the DEPC group, but it was significantly increased in the cypin group (*p* < 0.05), indicating that cypin could accelerate the swimming speed of zebrafish larvae. The spontaneous movement speed in the BPA treatment group and BPA + DEPC group both decreased significantly (*p* < 0.05), and that in the BPA + cypin group had no statistical difference compared to the BC group (*p* > 0.05), showing that BPA could suppress zebrafish movement speed and that cypin microinjection could reverse the BPA-induced movement speed inhibition of zebrafish larvae to normal levels.

**Figure 10:**
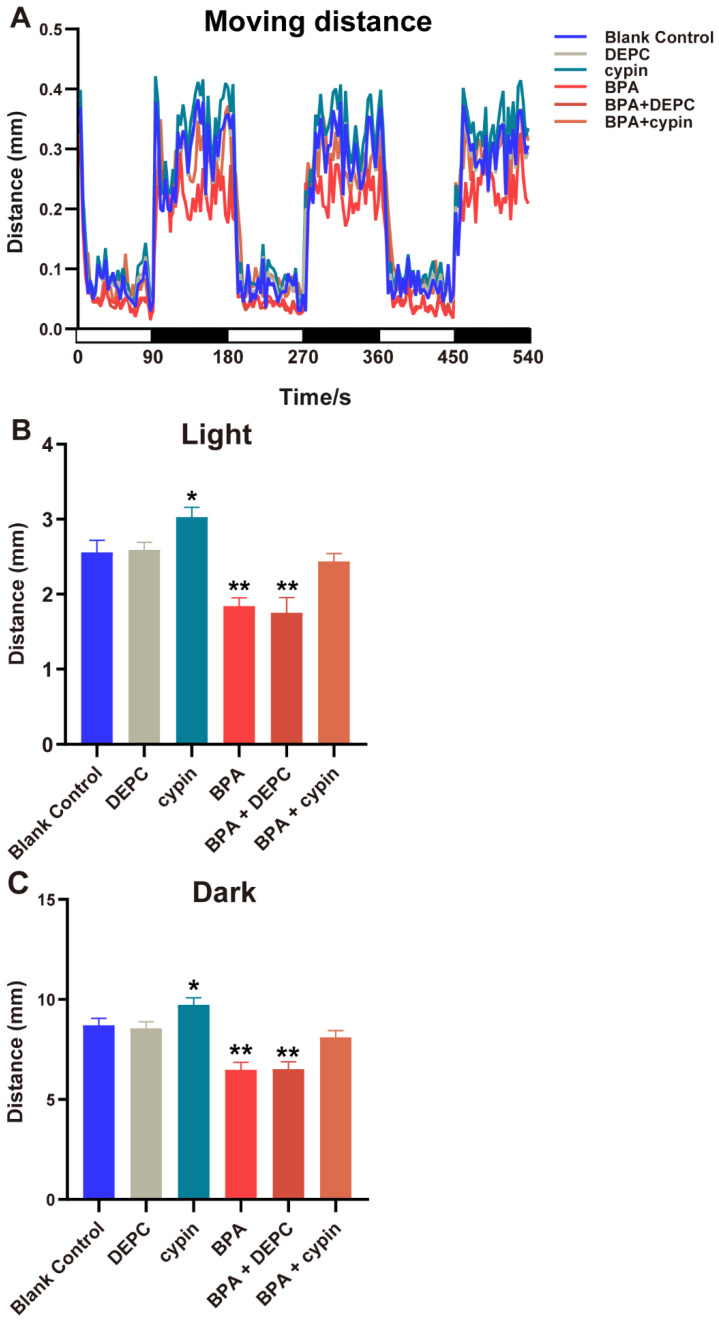
Effects of exposure to BPA on locomotor behavior under light-dark cycle stimulation of zebrafish embryos after microinjection of cypin mRNA. (**A**) The trend of distance. (**B**,**C**) The analysis results of the trend of the distance. Abscissa white for light, black for darkness. Data are the mean ± SD (n = 24 in each concentration). *, significant from blank control, *p* < 0.01; **, significant from blank control, *p* < 0.001.

The behavioral results of light and dark stimuli were consistent with the results of spontaneous movement speed (Figure 10A–C). After microinjection, the locomotor behavior of zebrafish larvae returned to normal level. All of the abovementioned results suggested that BPA mediates neurotoxicity and affects the locomotor behavior of zebrafish larvae by inhibiting the expression of cypin mRNA from the cleavage period to the segmentation period (1–24 hpf).

## 4. Discussion

Bisphenol A is an environmental endocrine disruptor. Scientists have reached the consensus that bisphenol A could be enriched in organisms through the food chain [29]. It is structurally similar to estrogen and competes for binding to estrogen receptors α/β, thus interfering with hormone signaling in the nervous system [30,31,32], which is essential for brain development. Prenatal exposure to BPA can impair the neurodevelopment of children, mainly manifested as anxiety, depression and hyperactivity [33,34].

In this study, the zebrafish larvae were exposed to BPA from the cleavage period to the segmentation period (1–24 hpf). Neurodevelopment in zebrafish begins during gastrula formation (approximately 6 hpf), when nerve cells arrive in place and form neural plates [35]. The neural plate becomes tubular at the end of gastrula formation (9–10 hpf), and different brain regions are segmented in the following 6–8 h [36]. At 24 hpf, zebrafish brain partitioning is basically complete, and the initial neurons are connected by axons [23,37]. Therefore, we deduce that the cleavage period to segmentation period (1–24 hpf) of zebrafish is a very important period of nervous system development. Our result did find that BPA exposure from the cleavage period to the segmentation period significantly decreased the spontaneous movement of zebrafish larvae. There was no difference in other periods. Locomotor behavior is a relatively sensitive indicator of internal physiological changes as well as of nerve injury. Thus, we believed that BPA induced neurotoxicity mainly in segmentation period. First, cypin, a key gene targeted by BPA, was upregulated during 1–24 hpf of development (Figure 6). Second, toxicokinetic prediction modeling showed that the concentration of BPA in zebrafish embryos (chorionated) gradually increased over time, reached the highest level at 40 hpf and then began to decrease. We found that the concentration of BPA in the prediction model increased continuously at 1–24 hpf, and our results showed that the expression of cypin only increased significantly at the 1–24 hpf period during the normal development of zebrafish embryos and decreased only at this period after BPA exposure. Therefore, even if the highest concentration is not reached in the 1–24 hpf period, it still produces the most serious effect. Therefore, we speculate that the toxicokinetics of BPA itself may play a role [38]. This modeling in our study correlates with our findings and suggests that the most susceptible period for BPA to exert its neurodevelopmental toxic effects is early in life, both in terms of its own toxicokinetics and the metabolic kinetics of zebrafish.

Cypin, known as cytosolic postsynaptic density protein 95 interactor, is also called the primary guanine deaminase (GDA; Guaninase) in the brain and plays key role in shaping neuronal circuits and regulating neuronal survival [39,40]. Microchemical analysis of the brain revealed that that cypin is mainly located in the dendritic axis and synaptic neck [39]. The overexpression of cypin in hippocampal neurons increases the number of dendrites, while knockdown could reduce the dendrite number [41] that is associated with GDA activity [41] and zinc ion-binding [42]. The decrease in the number of dendrites may lead to the failure of neurons to receive information normally, leading to neural abnormalities. Neuronal dendrites play an important role in the normal reception of information by the neural signal network. To understand the role of cypin in the neuron development of zebrafish, we tested cypin gene expression in different periods of embryonic development. The qPCR results showed cypin expression fluctuated with increasing exposure time compared to 1 hpf, increasing significantly at 24 hpf and falling back to the same level as 1 hpf at 48 hpf, 72 hpf and 96 hpf. The abovementioned results were consistent with the results of the Patel MV determination of the level of cypin in the mice brain, and the expression of cypin was increased at the beginning and then decreased to normal level [43]. Another study found that reduced locomotor behavior in children with autism spectrum disorder (ASD) may be due to cypin deficiency during pregnancy [44]. All of these findings indicate that cypin plays an important role in early neural development and are consistent with our findings.

To verify the role of cypin under BPA exposure, we detected the activity of guanine deaminase (GDA) and found that BPA could decrease the activity of GDA. GDA plays a critical role in neurodevelopment, and its enzyme activity is essential for normal purine recovery and brain development and function [41]. A specific reduction in guanine to adenine nucleotide levels was also found in Lesch-Nyhan disease, where a patient had dyspraxia and intellectual disabilities [45]. Our results did find that enzymes involved in purine metabolism, such as guanine deaminase, may play an important role in regulating appropriate neuronal activity, which suggests that the decrease of BPA-induced guanine deaminase activity may lead to impaired neurodevelopment in zebrafish. In summary, we speculate that BPA affects the neural development of zebrafish by interfering with the expression of cypin during the 1–24 hpf period. To confirm this hypothesis, we used a microinjection experiment of cypin mRNA. Then, microinjected embryos were exposed to BPA up to 24 hpf. The results of PCR showed that the level of cypin mRNA returned to normal, and the spontaneous locomotor behavior of zebrafish returned to the normal level. Therefore, we speculate that that cypin plays an important role in the neural development process from the cleavage period to the segmentation period (1–24 hpf) of zebrafish. Notably, based on the critical period identified in this study (1–24 hpf), there was a motor measurement endpoint directly related to the synapse, spontaneous caudal coiling, only at 24 hpf [46]. This would be a BPA neurodevelopmental endpoint that requires further attention in this study early in life.

The limitations and prospects of this study also need to be mentioned. It has been found that PSD-95 is involved in learning and memory impairment induced by BPA in rats [47], and the binding of cypin to PSD-95 correlates with the formation of stable dendrite branches [48]. Therefore, cypin may play a role in BPA-induced neurotoxicity by binding PSD-95 to affect the neural network. In addition, the study also found that RhoA, a member of the Rho family, has been shown to regulate dendritic crystal formation, global dendritic structure and dynamic dendritic behavior [49], and activated RhoA acts as a negative regulator of dendritic branching by reducing cypin expression in a translocation-dependent manner [50]. Does BPA affect the expression of RhoA, thereby regulating the expression of cypin and thus affecting the locomotor behavior of zebrafish? Further discussion and confirmation of the mechanism are still needed in the future.

## 5. Conclusions

In this study, we concluded that the sensitive period of BPA to neurotoxicity in zebrafish embryos is from the cleavage period to the segmentation period (1–24 hpf), which is manifested as the inhibition of locomotor behavior in zebrafish. Moreover, BPA can mediate neurotoxicity by downregulating the expression of cypin mRNA from the cleavage period to the segmentation period (1–24 hpf) and influence the locomotor behavior of zebrafish. Therefore, we speculate that cypin gene may be a potential biomarker of neural development.

## Data Availability

All data generated or analyzed during this study are included in this published article (and its Supplementary Information Files).

## Supplementary Materials

The following supporting information can be downloaded at: https://www.mdpi.com/article/10.3390/toxics11020177/s1, Table S1: Primer sequences for target genes.
