# Supplementary material for: Stage-Related Neurotoxicity of BPA in the Development of Zebrafish Embryos
Source: Toxics. 2023 Feb 14;11(2):177. doi: 10.3390/toxics11020177 (PMC9963847; doi:10.3390/toxics11020177)
Supplement: Supplementary file 1 [file toxics-11-00177-s001.zip › toxics-2164641-supplementary.pdf]

Supporting Information

Table S1. Primer sequences for target genes.

Gene Name	Primer Sequence (5'-3')
β-actin	F: ACGAACGACCAACCTAAACTCT
	R: TTAGACAACTACCTCCCTTTGC
cypin	F: TGATGCCTCACTTCTGCTCG
	R: CCATGCAAACTTTCCCCACT
